# High-Voltage Flexible Aqueous Zn-Ion Battery with Extremely Low Dropout Voltage and Super-Flat Platform

**DOI:** 10.1007/s40820-020-0414-6

**Published:** 2020-03-19

**Authors:** Zhe Chen, Panpan Wang, Zhenyuan Ji, Hua Wang, Jie Liu, Jiaqi Wang, Mengmeng Hu, Yan Huang

**Affiliations:** 1 State Key Laboratory of Advanced Welding and Joining, Harbin Institute of Technology, Shenzhen, 518055 People’s Republic of China; 2 Flexible Printed Electronic Technology Center, Harbin Institute of Technology, Shenzhen, 518055 People’s Republic of China; 3 School of Materials Science and Engineering, Harbin Institute of Technology, Shenzhen, 518055 People’s Republic of China

**Keywords:** Flexible, Aqueous zinc-ion battery, High voltage, Flat platform, Rate capability

## Abstract

Flexible rechargeable aqueous zinc-ion batteries (ZIBs) have attracted extensive attentions in the energy storage field due to their high safety, environmental friendliness, and outstanding electrochemical performance while the exploration of high-voltage aqueous ZIBs with excellent rate capability is still a great challenge for the further application them in flexible and wearable electronics. Herein, we fabricated a 2.4 V high-voltage flexible aqueous ZIB, being among the highest voltage reported in aqueous ZIBs. Moreover, it exhibits extremely flat charging/discharging voltage platforms and the dropout voltage is only 0.1 V, which is the smallest gap in all aqueous batteries to our best knowledge. Furthermore, the prepared ZIB performs high rate capability of 25 C and energy density of 120 Wh kg^−1^ and exhibits excellent safety under various destructive conditions including hammering, sewing, punching, and soaking. These extraordinary results indicate the great application potential of our high-voltage flexible aqueous ZIB in wearable electronics.

**Electronic supplementary material:**

The online version of this article (10.1007/s40820-020-0414-6) contains supplementary material, which is available to authorized users.

## Highlights

- A 2.4 V high-voltage flexible aqueous ZIB was fabricated, and superiors performances were achieved: extremely flat charging/discharging voltage plateaus (1.9/1.8 V), the smallest plateau voltage gap of 0.1 V, high energy density of 120 Wh kg^−1^, high power density of 3700 W kg^−1^, and excellent rate capability of 25 C.
- The battery posed application potential in wearable electronics with extreme safety against tough destructions including hammering, sewing, punching, and soaking.

**Electronic supplementary material:**

The online version of this article (10.1007/s40820-020-0414-6) contains supplementary material, which is available to authorized users.

## Graphical abstract

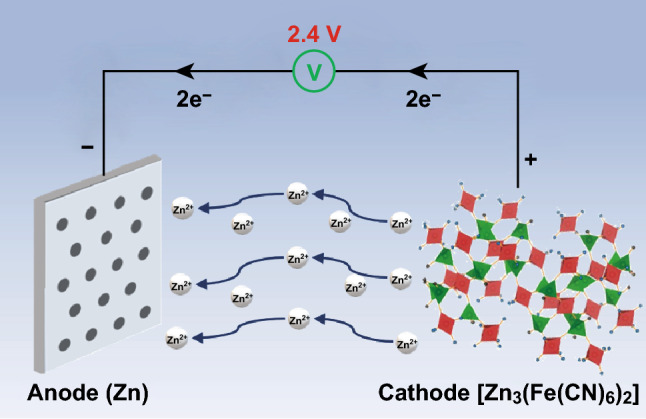

## Introduction

With the increasing demand for flexible electronic devices such as smart bracelet, flexible sensor, and smart clothing, more stringent requirements are raised for the energy storage devices including high voltage, high energy density, environmental friendliness, favorable mechanical property, etc. [1–5]. Although commercial lithium-ion batteries are the mainstream power source in the energy storage market, their visible defects such as the restrictive lithium resources, complicated assembly process, and especially for the toxic organic electrolytes, prevent their further wide application in the smart and wearable electronics [6–11]. In this regard, aqueous rechargeable zinc-based batteries (ZIBs) possess the overwhelming advantages in terms of unexceptionable safety and non-toxicity feature. Moreover, zinc was regarded as an ideal anode for aqueous metal ion batteries because of its low potential (− 0.78 V vs. SHE) and high theoretical capacity of 820 mAh g^−1^ [12, 13]. However, the output voltage of ZIBs was still far from satisfactory since the voltage of the most common zinc–manganese battery is not higher than 1.5 V, which was mainly attributed to the lack of high-voltage cathode material and narrow electrochemical stability window of liquid aqueous electrolyte for the high valence and small radius of zinc ion [14, 15]. In this regard, exploitation of high-voltage ZIBs with appropriate cathode material and electrolyte system is imperative to fulfill the power requirement for various wearable electronics.

In addition, there are two vitally important issues that have been ignored in many aqueous battery reports: One is the stability of charging and discharging platform, and the other is the dropout voltage between charging and discharging platform. The former is critical to the power output and energy density for energy storage device, while the latter determines the energy efficiency of the battery [16–19]. However, most of the reported aqueous batteries, which have no visible platform for charging and discharging, present constantly changing voltage and even display similar triangular curves to the super-capacitors [20, 21]. And in these very few aqueous batteries possessing charge/discharge platforms (such as Zn//MnO_2_ and Zn//Ag), the dropout voltage is obviously higher than 0.1 V, followed their relatively low voltage and high cost of noble metal (Ag) as well [22, 23]. Thus, the exploration of ZIBs with super-flat platform, 0.1 V dropout voltage, and low cost is extremely appealing for the further widespread application of high-voltage ZIBs in high power electronics.

Inspired by ultra-high ionic conductivity of gelatin-g-PAM gel electrolyte, minor polarization open-framework structure of zinc hexacyanoferrate (ZnHCF) and low potential of zinc [14, 24], a flexible aqueous Zn-ion full battery was fabricated here based on Zn^2+^ intercalation and deintercalation with 2.4 V high voltage, super-flat platform, and dinky 0.1 V dropout voltage for charging/discharging. To the best of our knowledge, this high voltage, extremely flat platform, tiny dropout of ZIB in aqueous system is rarely reported. Moreover, the ZIB in our work performs excellent rate capability of 25 C and energy density of 120 Wh kg^−1^. Batteries light up long light strips and demonstrate extraordinary security against various destruction including hammering, stitching, punching and soaking, posing great application prospects in the next-generation wearable and smart electronic devices.

## Experimental Section

### Synthesis of Polyelectrolyte

The hierarchical polymer electrolyte (HPE) was synthesized by in situ free radical polymerization. Detailedly, 7.27 g zinc trifluoromethanesulfonate (Zn(CF_3_SO_3_)_2_, 98%, Macklin) was fully dissolved into 20 mL DI water at room temperature. Then, 2 g gelatin (photographic grade, Aladdin) and 30 mg potassium persulfate (KPS) were added in turn to the solution, which were stirred slowly at 80 °C to accelerate dissolution and avoid bubbles. After cooling down to 40 °C, 3 g acrylamide (AM, AR grade, Macklin) and 3.5 mg *N*,*N*′-methylenebisacrylamide (BIS, CP grade, Aladdin) were added into the mixture solution ordinally [14]. After stirring 2 h for grafting reaction, the above solution was injected into a mold with a tiled polyester membrane, which was transferred into an oven at 60 °C for 2–3 h. Finally, a translucent 1 M Zn(CF_3_SO_3_)_2_ HPE with grafted structure was obtained. As a contrast, gelatin electrolyte (GE) film was obtained by the same way except for the addition of acrylamide and *N*,*N*′-methylenebisacrylamide.

### Fabrication of Electrodes and Battery Assembly

The cathode material was prepared by high-temperature coprecipitation. Typically, 100 mL of 0.1 M ZnSO_4_ and 0.05 M K_3_Fe(CN)_6_ mixed aqueous solution was added dropwisely into 50 mL DI water at 60 °C [25]. Titration was kept at a constant rate until precipitation was obtained. After stirring strongly for 1 h to allow sufficient reaction, the product was rinsed with DI water and centrifuged at 6000 rpm for 2 min for several times to remove the residues. Then, the washed sediment was placed in a vacuum oven and dried at 70 °C for 24 h to obtain the cathode active material ZnHCF. At last, the active material (ZnHCF), conductive agent (carbon nanotubes) and binder (polyvinylidene fluoride, PVDF) were mixed at a mass ratio of 8:1:1 with moderate *N*-methyl pyrrolidone (NMP) as solvent, vigorously milled for more than 5 h and painted onto the carbon cloth for 10 h drying at 60 °C.

For the Zn anode, the carbon cloth was placed into ethanol under ultrasonic operation for more than 15 min before electrodeposition. Then, the hydrophilic carbon cloth was inserted into 1 M zinc sulfate (ZnSO_4_) solution as the working electrode. Thus, a Zn anode was acquired by anodic electrodeposition at − 0.8 V in the two-electrode system with fairly sized Zn sheet as cathode.

For the full cell assembly, the as-prepared cathode, gel electrolyte, and Zn anode were packed together under the ambient condition to form a compacted and sandwiched structure for electrochemical performance and security tests.

### Materials Characterization and Electrochemical Performance Test

The structural information of the as-prepared electrode materials was collected by X-ray diffraction [XRD, Bruker D2 Phaser diffractometer with Cu K*α* irradiation (*λ* = 1.54 Å)] and Raman (Renishaw Invia Reflex system (UK), with an excitation wavelength of 514 nm). The morphology of powder active samples and deposited zinc was characterized by field scanning electron microscope (FE-SEM, FEI/Philips XL30). The polymer structure analysis was employed by Fourier transform infrared spectroscopy (FTIR). For electrochemical testing, electrochemical impedance spectroscopy (10 kHz to 0.01 Hz), cyclic voltammetry, and galvanostatic charge/discharge measurements in gradient current density were obtained via an electrochemical workstation (CHI 760e). The electrochemical cycling test was carried out by battery testing system (LANHE, CT2001A).

## Results and Discussion

**Fig. 1:**
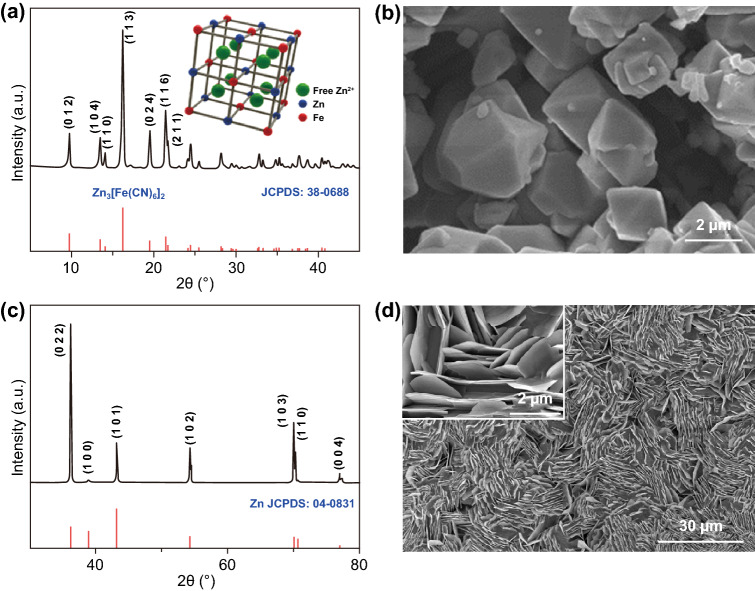
**a** XRD patterns of the obtained cathode material and standard card (JCPDS No. 38-0688). Inset is the open-framework atomic configuration model. **b** SEM image of ZnHCF powder. **c** XRD patterns of the electrodeposited Zn and standard card (JCPDS No. 04-0831). **d** Low-magnification SEM image of the electrodeposited Zn and high-magnification inset in it

The structure of the obtained cathode material was analyzed by XRD as shown in Fig. 1a. In the XRD pattern, all the diffraction peaks are well indexed to the rhombohedral Zn_3_[Fe(CN)_6_]_2_ (ZnHCF, JCPDS No. 38-0688) with three main diffraction peaks located around 16.2°, 19.5°, and 21.5°, which belong to the crystal plane diffraction of (113), (024), and (116), respectively. To make a clear understanding on the crystal structure of ZnHCF, the atomic framework configuration model was given in the inset in Fig. 1a. The Prussian blue analogs ZnHCF is a face-centered cubic framework with the transition metal cations Zn and Fe coordinated by CN ligands [26–29]. The formed 3D framework contains large channels and interstitial sites to allow facile mobility of free Zn^2+^ ions. Further, the Raman spectrum was utilized to investigate valence state of transition metal Fe cation as shown in Fig. S1. The wavenumber peak of ν(CN) located at 2201 cm^−1^ corresponds to the stretching vibration mode of the cyanide CN^−^ that coordinated to Fe(III), which is in good accordance with the XRD analysis [30]. From the SEM image of ZnHCF (Fig. 1b), we can find that the ZnHCF grain exhibits rhombohedral morphology. Meanwhile, the XRD pattern and SEM image of electrodeposited zinc are displayed in Fig. 1c, d. As shown in Fig. 1c, the XRD peaks of Zn match well with the standard diffraction peaks of Zn metal (JCPDS No. 04-0831) [1]. From Fig. 1d, it can be seen that the Zn was uniformly distributed and highly aligned on the carbon cloth substrate. A small area is selected for higher-magnification enlargement, and the clear image conveys a sheetlike laminated structure perpendicular to the surface of the substrate which maximizes the area of zinc and provides a large specific surface.

**Fig. 2:**
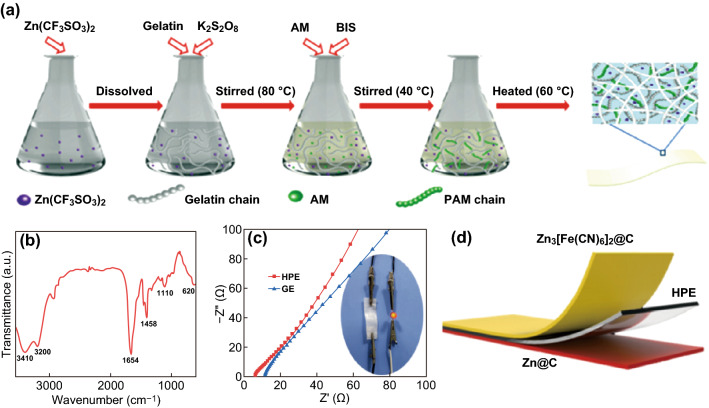
**a** Synthesis route scheme of HPE electrolyte. **b** FTIR spectrum of the gelatin-g-PAM HPE treated by lyophilization. **c** EIS comparison of HPE and pristine GE in the frequency range from 10 kHz to 0.01 Hz, attached is the demonstration of electrolyte conduction. **d** Schematic illustration of the flexible aqueous ZIB with sandwich structure

Meanwhile, the synthesis route schematic diagram of HPE is provided in Fig. 2a. Herein, the K_2_S_2_O_8_ was the initiator, and BIS was the grafting agent with AM monomer to form branch on gelatin chain. By this way, a highly cross-linked HPE hydrogel polymer film was obtained. FTIR (Fig. 2b) is used to certify the gelatin-g-PAM structure of HPE. There exist several distinct absorption peaks in the spectrum including 3410 cm^−1^ (symmetric N–H stretching vibration) and 3200 cm^−1^ (antisymmetric N–H stretching vibration) of primary amine, 1654 cm^−1^ (amide I, C=O stretching vibration), 1458 cm^−1^ (C–N stretching vibration), 1110 cm^−1^, and 620 cm^−1^ (amino oscillating peak), respectively [31]. The abundance of amide bonds contributes to the excellent water retention and outstanding viscosity which causes the electrode to stick tightly to the electrolyte. Meanwhile, the ionic conductivity of the gel electrolyte was calculated based on EIS analysis, and high ionic conductivities of 2.04 × 10^−3^ S cm^−1^ for HPE and 1.09 × 10^−3^ S cm^−1^ for GE were obtained as indicated in Fig. 2c. We cut the polymer electrolyte with a block area of 1 × 1 cm^2^ followed clamped by stainless steel plate to test impedance, which was fully compact and stood for a while to exhaust the air in the gap while stabilizing the system. Then, we measured its thickness, obtained the resistance from figure, and calculated it according to the formula *σ* = *l*/*SR* (*σ* is the ionic conductivity; *l* is thickness; *S* is the contact area of the electrolyte; *R* is the resistance). Comparison data of several common zinc-ion gel electrolytes are displayed in Table S1 [14]. As an intuitive demonstration of their good ionic conductivity, the HPE electrolyte could be connected in an electronic circuit to light up a LED bulb as shown in the inset in Fig. 2c. Thus, a flexible aqueous rechargeable zinc-ion battery was acquired with the sandwiched structure as shown in Fig. 2d, which was assembled by Zn as anode, ZnHCF as cathode, and HPE as electrolyte, respectively.

**Fig. 3:**
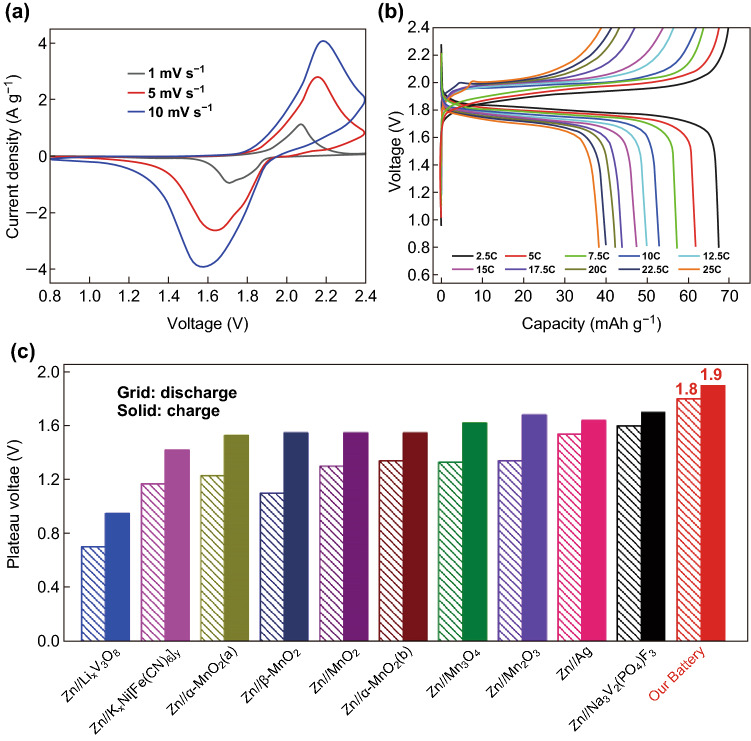
**a** CV curves of the ZIB at different scan rates of 1 mV s^−1^ (black), 5 mV s^−1^ (red) and 10 mV s^−1^ (blue). **b** Charge/discharge profiles from 2.5 to 25 C (1 C = 80 mA g^−1^). **c** Plateau voltage comparison of various ZIBs in previous reports. (Color figure online)

A sequence of measurements was performed to explore the electrochemical behaviors of ZIB. The cyclic voltammetry profiles between the voltage window of 0.8 and 2.4 V at different scan rates are provided in Fig. 3a. As shown, a pair of strong redox peaks located around 1.6/2.2 V was observed, which was correlated with the conversion of [Fe^III^(CN)_6_]^3−^↔[Fe^II^(CN)_6_]^4^ and Zn ↔ Zn^2+^. During the cathodic process, the distinct reduction peak around 1.57 V (scan rate of 10 mV s^−1^) was related to the transformation of [Fe^III^(CN)_6_]^3−^ to [Fe^II^(CN)_6_]^4−^ and the Zn dissolution. While during the anodic process, the oxidization peak at 2.19 V was accompanied by the oxidation of Fe^II^ to Fe^III^ and the precipitation of Zn^2+^ to Zn metal. The electrochemical reaction equation of the full ZIB could be illustrated as Eqs. (1) and (2) [5, 32–34]:

1 $$2\left[ {{\text{Fe}}\left( {\text{CN}} \right)_{6} } \right]^{3 - } + {\text{Zn}}\mathop \to \limits^{\text{discharge}} 2\left[ {{\text{Fe}}\left( {\text{CN}} \right)_{6} } \right]^{4 - } + {\text{Zn}}^{2 + }$$

2 $$2\left[ {{\text{Fe}}\left( {\text{CN}} \right)_{6} } \right]^{4 - } + {\text{Zn}}^{2 + } \mathop \to \limits^{\text{charge }} 2\left[ {{\text{Fe}}\left( {\text{CN}} \right)_{6} } \right]^{3 - } + {\text{Zn }}$$

Figure 3b exhibits the galvanostatic charge/discharge profiles in the voltage window of 0.8–2.4 V at various rates ranging from 2.5 to 25 C. Excitingly, it displayed extremely high and flat charge/discharge plateaus located around 1.9/1.8 V with the plateau voltage difference of merely 0.1 V at 2.5 C. It is worthy to mention that the excellent voltage plateau performance with high output voltage is the best of all aqueous ZIBs reported to our best knowledge. Most of the reported aqueous batteries have no visible platform for charging/discharging and even exhibit triangular curves like super-capacitors [35–37]. Furthermore, in these very few aqueous batteries, which possess relatively stable charge/discharge platforms such as Zn//MnO_2_ and Zn//Ag, their average platform voltage is no more than 1.7 V, while the dropout voltage is obviously higher than 0.1 V [22, 23]. The detailed comparison of various ZIBs reported in previous works is presented in Fig. 3c, in which many other ZIBs with low voltage and without charge/discharge plateaus are not included [20, 21, 35–49]. Obviously, the discharging plateau voltage of our ZIB is the highest among ZIBs, such as Zn//Li_*x*_V_3_O_8_ (0.7 V), Zn//K_*x*_Ni[Fe(CN)_6_]_*y*_ (1.17 V), Zn//*α*-MnO_2_(a) (1.23 V), Zn//*β*-MnO_2_ (1.1 V), Zn//MnO_2_ (1.3 V), Zn//*α*-MnO_2_(b) (1.34 V), Zn//Mn_3_O_4_ (1.33 V), Zn//Mn_2_O_3_ (1.34 V), Zn//Ag (1.54 V), and Zn//Na_3_V_2_(PO_4_)F_3_ (1.6 V). The high ionic conductivity of PAM gel electrolyte and the excellent (de)intercalation ability of ZnHCF contribute greatly to the 1.8 V high discharging platform of our battery. Moreover, our charging/discharging plateau voltage gap of 0.1 V is the lowest, e.g., Zn//Li_*x*_V_3_O_8_ (0.25 V), Zn//K_*x*_Ni[Fe(CN)_6_]_*y*_ (0.25 V), Zn//*α*-MnO_2_(a) (0.3 V), Zn//*β*-MnO_2_ (0.45 V), Zn//MnO_2_ (0.25 V), Zn//*α*-MnO_2_(b) (0.21 V), Zn//Mn_3_O_4_ (0.29 V), Zn//Mn_2_O_3_ (0.34 V), Zn//Ag (0.1 V), Zn//Na_3_V_2_(PO_4_)F_3_ (0.1 V). The small electrochemical polarization during charge/discharge process is mainly benefited from the superior ionic conductivity of HPE as well as the favorable ionic transport pathway in the open framework in the ZnHCF cathode, which contributes to the excellent electrochemical performance of our ZIB. We investigated the effect in terms of various zinc salts. Three different zinc salts (Zn(CF_3_SO_3_)_2_, ZnSO_4_, and Zn(CH_3_COO)_2_) were selected to analyze the nature of electrolyte under the same concentration and electrode parameters. CV and GCD were carried in each solution shown in Figs. S2 and S3. From the CV curves, Zn(CF_3_SO_3_)_2_ presented the highest discharge voltage than ZnSO_4_ and Zn(CH_3_COO)_2_. GCD plots gave the further demonstration for platform performance and capacity. Combined with ionic conductivity data (Table S1), in ZnHCF crystals, whether the velocity of Zn^2+^ intercalation/extercalation can keep up with the change of Fe^2+^ valence mainly determines the final electrochemical performance. In order to explore the most suitable electrolyte concentration for better performance, an inquiry experiment was designed and the result is shown in Fig. S4. Considering the solubility of Zn(CF_3_SO_3_)_2_ is 3.7 M, we selected four concentration gradients (0.5, 1, 2.5, and 3.7 M) to perform CV tests at 10 mV s^−1^ under as same battery conditions as possible. The results show that 1 M is the most suitable electrolyte concentration. A significant large polarization emerges at 0.5 M; the voltage performance at 2.5 M is still not ideal; 3.7 M is the closest to 1 M, but the performance in the high-voltage range is not as good as 1 M, showing a clear oxygen evolution trend.

**Fig. 4:**
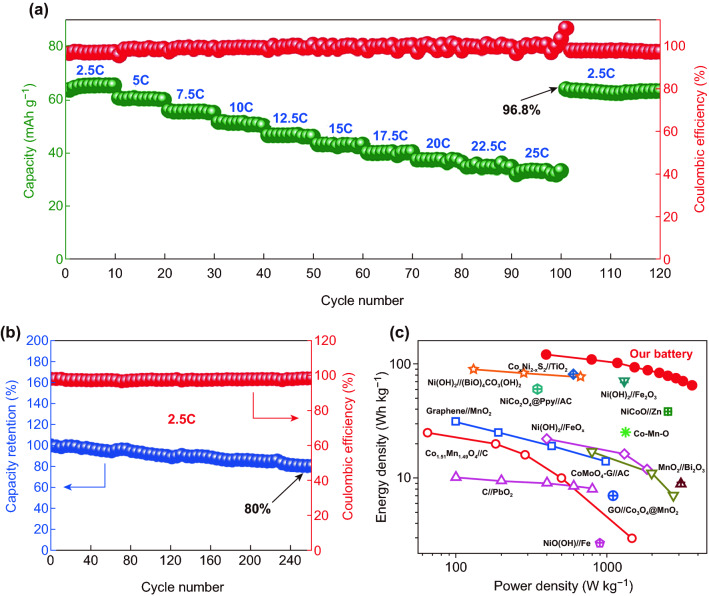
**a** Rate capability of the ZIB ranging from 2.5 to 25 C. **b** Capacity retention and corresponding coulombic efficiency of ZIB upon long cycles. **c** The Ragone plot of our work with others reported in previous works

Additionally, the rate capability is revealed in Fig. 4a under ten gradient current densities from 200 to 2000 mA g^−1^. The ZIBs could deliver a specific capacity of 67, 62, 57, 53, 50, 47, 44, 42, and 40 mAh g^−1^ at 2.5, 5, 7.5, 10, 12.5, 15, 17.5, 20, and 22.5 C rate, respectively. At the minimum current density 2.5 C, the sufficient electrode response makes the capacity of the battery relatively high, accounting for 77.9% of the calculated theoretical capacity (86 mAh g^−1^). A high capacity of 38 mAh g^−1^ could be maintained even under the high rate of 25 C. When the current rate returned back to 2.5 C, the capacity retention as high as 96.8% was exhibited with the coulombic efficiency over 97% during cycles, further implying the excellent rate capability of the ZIB. Besides, the long cyclic stability was pictured in Fig. 4b at the current rate of 2.5 C, which displayed 80% capacity retention after 260 cycles. Compared with previous studies Zn//ZnSO_4_//Zn_3_V_2_O_7_(OH)_2_·2H_2_O (68% capacity retention after 300 cycles) and Zn//ZnSO_4_//Zn_3_[Fe(CN)_6_]_2_ (80% capacity retention after 200 cycles), our ZIB shows distinctly better cycling stability [30, 50]. In spite of this, they are still a big weakness and not as good as Mn-based and V-based zinc-ion batteries. Benefited by the high operating voltage, our ZIB delivers high energy density of 120 Wh kg^−1^ and high power density of 3700 W kg^−1^, respectively, which are much superior than many other battery systems supported by previous references, including Zn//NiCoO, Gr//MnO_2_, etc., as described in the diagram in Fig. 4c [24, 51–67]. In general, our high-voltage ZIB exhibits superior charge/discharge voltage plateaus and high energy and power density, demonstrating great application prospect in flexible and wearable electronics.

**Fig. 5:**
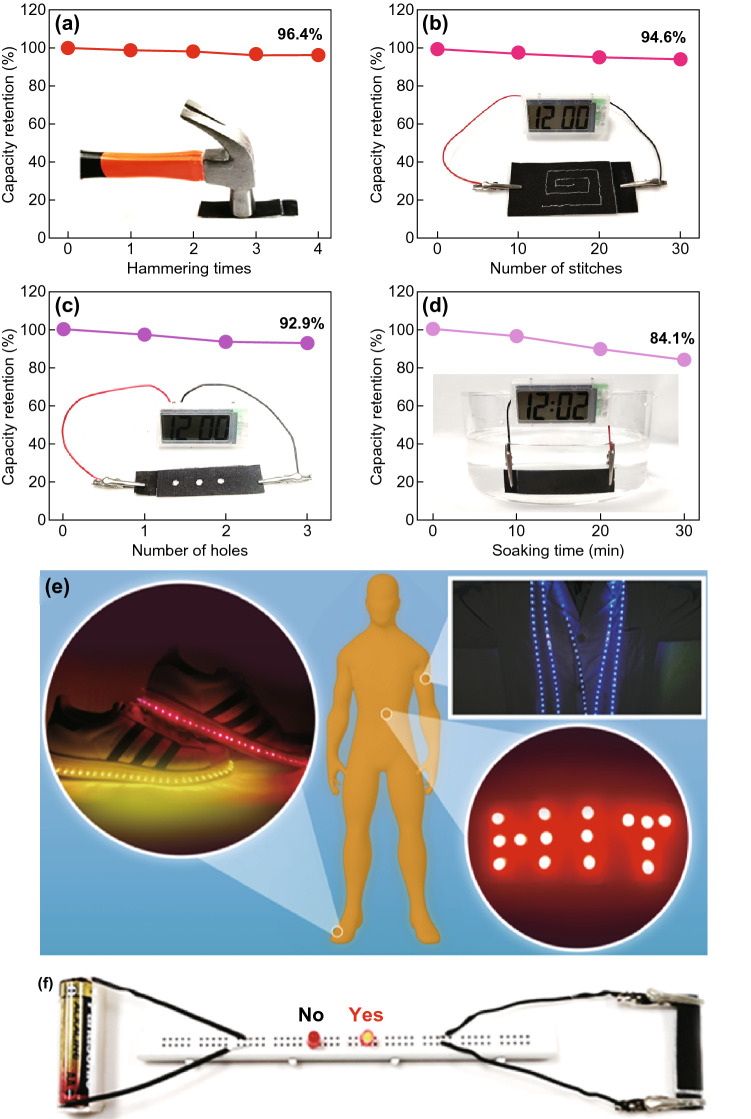
Electrochemical performance of the ZIB after **a** hammering, **b** sewing, **c** punching, and **d** soaking test. **e** Various LED patterns (HIT, luminous shoes, light clothes) powered by designed batteries and visual voltage contrast demo. **f** The visual contrast demo consists of a standard voltaic battery and a small ZIB (1 × 4 cm^2^)

Considering the harsh requirements of flexible and wearable electronics, various tough environments including hammering, sewing, punching, and soaking are simulated to investigate the electrochemical performance of our ZIB under destructive conditions. Nevertheless, batteries composed of only electrode and electrolyte in a sandwich structure are also excellent in flexibility, and it can be bent freely even under a thick protective film in Fig. S5. In order to explore the effect of external forces, we continuously hammered the battery to monitor its capacity variation (Fig. 5a). The capacity retention could reach 96.4% after five times of fierce hammering (the external force was estimated to be at least 26.3 kPa). Moreover, the ability to be sewable is very essential for the wearable application as it is often used in the situation of integration with cloths. By using homely hand sewing, over 30 stitches were acted on the full battery and each pinhole is about 0.5 mm. The capacity was tested every ten shots which exhibits a rather high capacity retention of 94.6% after 30 stitches (Fig. 5b). Furthermore, the artificial punching was adopted to investigate the resistance against damage (Fig. 5c). We used a standard-sized puncher (*d* = 3 mm) to drill holes in the battery. A high capacity retention of 92.9% was obtained after three holes were punched. In addition to hammering, sewing, and punching, soaking is also a common application scenario for wearable electronics like washing, rain, sweat, etc. Without any waterproof treatments, we sewed the battery, soaked it into the water, and took it out every 10 min for testing. As shown in Fig. 5d, the capacity remained 84.1% after 30 min of soaking. These results further verified that our flexible ZIB exhibits excellent tolerance against demanding situation for wearable devices. Furthermore, a ZIB with size of 4 × 6 cm^2^ could easily light up a HIT pattern comprised of 15 LEDs in parallel. Meanwhile, several batteries connected and paralleled together can brighten the long luminous light strip laid in shoes and clothes as shown in Fig. 5e. To make a more intuitive expression of the high-voltage ZIB, we provided a visual contrast consisted of a standard voltaic battery (size: 1.4 × 5 cm^2^; mass: 25 g) and our ZIB (size: 1 × 4 cm^2^; mass: 0.4 g) in Fig. 5f. The small ZIB can easily light the LED bulb while the disposable battery failed, which fulfills the requirements of lightweight and high voltage for flexible and wearable devices. These successful lightings further confirm the potential application of our high voltage, high energy density, and safe ZIB in future wearable and smart electronics.

## Conclusions

In summary, we develop a 2.4 V high-voltage flexible aqueous Zn//ZnHCF battery which delivers high rate capability and excellent platform performance. The smallest dropout voltage of 0.1 V for the extremely flat charging/discharging voltage platforms is realized unprecedentedly to our best knowledge. Meanwhile, the great energy density and power density of 120 Wh kg^−1^ and 3700 W kg^−1^, respectively, with 25 C high rate capability of the ZIB in our work have considerable application prospect in energy fields. Furthermore, it is demonstrated to be reliable energy storage which possesses excellent safety and capacity retention under various destructive conditions, strongly demonstrating the promising application of our high-voltage flexible ZIB in future wearable and smart electronics.

## Electronic supplementary material

Below is the link to the electronic supplementary material.

Supplementary material 1 (PDF 274 kb)
