# Supplementary material for: High-Voltage Flexible Aqueous Zn-Ion Battery with Extremely Low Dropout Voltage and Super-Flat Platform
Source: Nanomicro Lett. 2020 Mar 19;12:75. doi: 10.1007/s40820-020-0414-6 (PMC7770716; doi:10.1007/s40820-020-0414-6)
Supplement: Supplementary file 1 — Supplementary material 1 (PDF 274 kb) [file 40820_2020_414_MOESM1_ESM.pdf]

Supporting Information for

High-Voltage Flexible Aqueous Zn-Ion Battery with Extremely Low Dropout Voltage and Super-Flat Platform

Zhe Chen[1, 2, 3, #], Panpan Wang[1, 2, 3, #], Zhenyuan Ji[1, 2, 3], Hua Wang[1, 2, 3], Jie Liu[1, 2, 3], Jiaqi Wang[1, 2, 3], Mengmeng Hu[1, 2, 3], Yan Huang[1, 2, 3, *]

[1]State Key Laboratory of Advanced Welding and Joining, Harbin Institute of Technology, Shenzhen 518055, People's Republic of China

[2]Flexible Printed Electronic Technology Center, Harbin Institute of Technology, Shenzhen 518055, People's Republic of China

[3]School of Materials Science and Engineering, Harbin Institute of Technology, Shenzhen 518055, People's Republic of China

[#]Zhe Chen and Panpan Wang contribute equally to this work

*Corresponding author. E-mail: yanhuanglib@hit.edu.cn (Yan Huang)

Supplementary Table and Figures

Table S1 The ionic conductivity comparison of common zinc ion gel electrolytes

Gel types	Ionic conductivity mS cm^{-1}	References
HPE	2.04	This work
GE	1.09	This work
PEO	0.5~0.8	[S1]
PEO	2~4	[S2]
Poly-ε-caprolactone	0.88	[S3]
Poly(4-vinylpyridine)	2×10^{-5}	[S4]

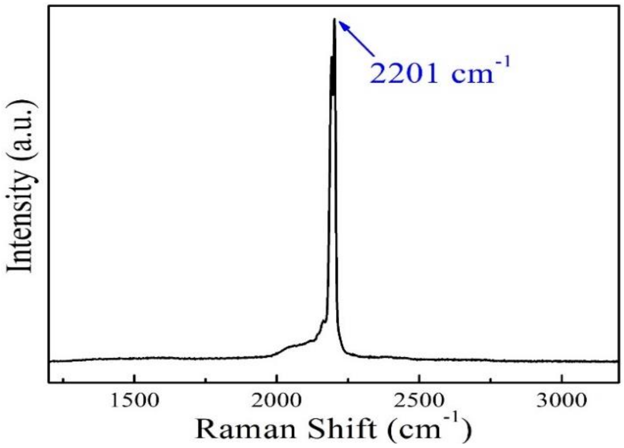

Fig. S1 Raman spectrum of ZnHCF active materials

The wavenumber peak of ν(CN) located at 2201 cm^{-1} corresponds to the stretching vibration mode of the cyanide CN^- that coordinated to Fe(III), which accords with the structure of ZnHCF. [S5].

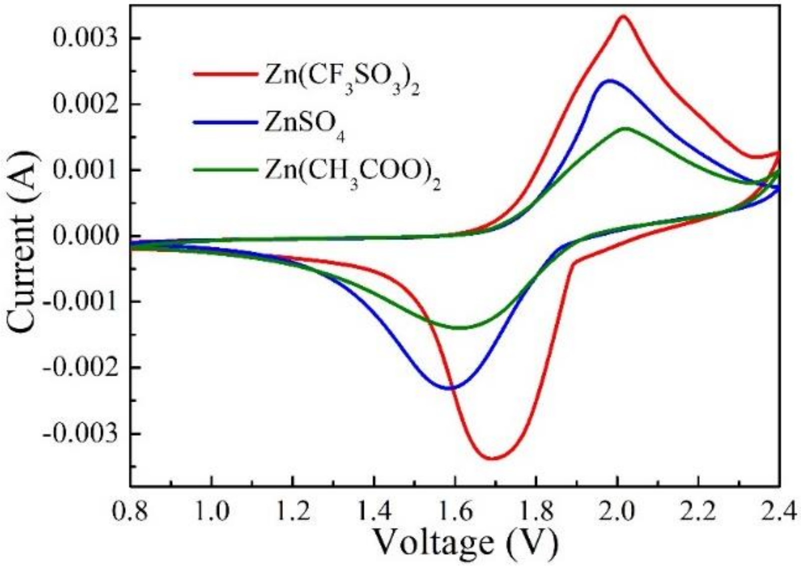

Fig. S2 CV curves of three zinc salts ($Zn(CF_3SO_3)_2$, $ZnSO_4$, $Zn(CH_3COO)_2$) at 5 mV s^{-1} scan rate

In the picture, the charging/discharging voltage 2.01/1.69 V of $Zn(CF_3SO_3)_2$, 1.98/1.58 V of $ZnSO_4$, 2.01/1.62 V of $Zn(CH_3COO)_2$ give a distinct result that $Zn(CF_3SO_3)_2$ is more suitable for electrolytes of ZIBs to give full play to their high voltage performance.

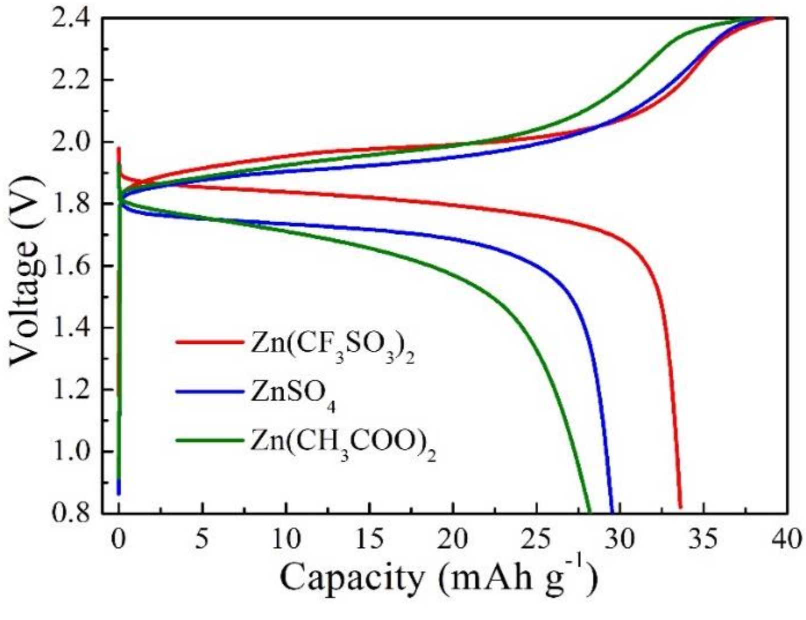

Fig. S3 GCD plots of three zinc salts ($Zn(CF_3SO_3)_2$, $ZnSO_4$, $Zn(CH_3COO)_2$) at 12.5 C

$Zn(CF_3SO_3)_2$ shows the highest capacity and excellent voltage platform, as well as the smallest dropout voltage among the three salts.

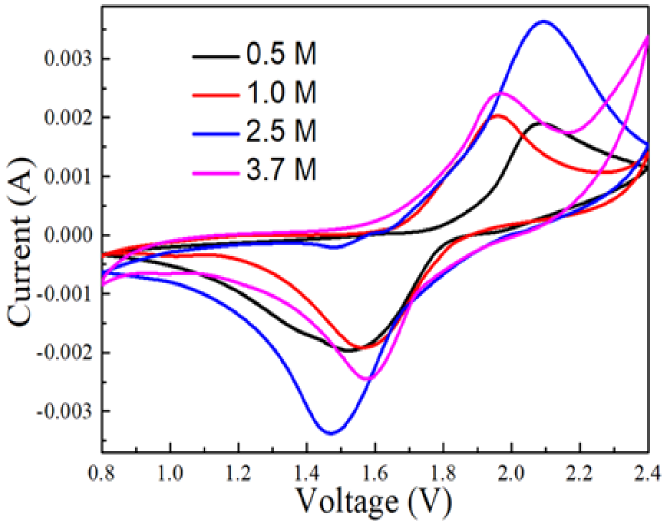

Fig. S4 CV curves of four $Zn(CF_3SO_3)_2$ concentrations

Four concentration gradients (0.5 M、1 M、2.5 M、3.7 M) were selected to perform CV tests at 10 mV s^{-1} under as same battery conditions. The results show that 1 M is significantly better than other concentrations.

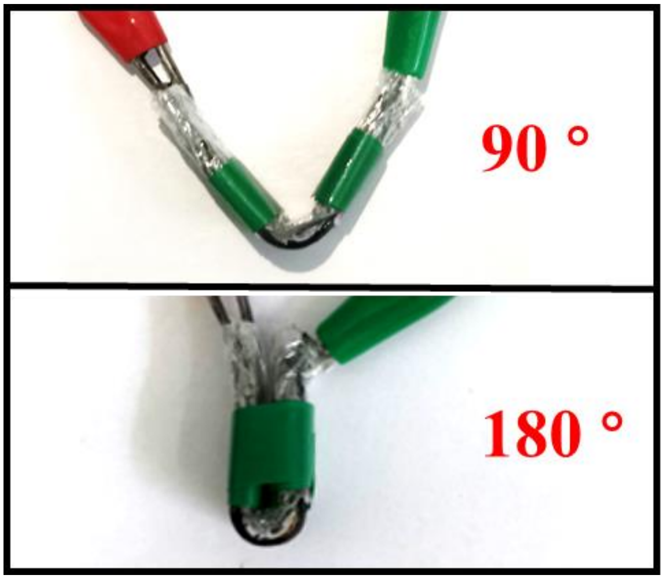

Fig. S5 Images of the battery at different bending angles

Batteries assembled in sandwich structures bent at 90° and 180° respectively, which shows the electrode is in good contact with the electrolyte. Despite being covered with a thick protective film, the battery still maintains good flexibility.

Supplementary References

[S1] I. Pucić, A. Turković, Radiation modification of $(PEO)_8$-$ZnCl_2$ polyelectrolyte and nanocomposite. Solid State Ionics **176**(19-22), 1797-1800 (2005). https://doi.org/10.1016/j.ssi.2005.04.042

[S2] A. Turković, M. Pavlović, P. Dubček, M. Lučić-Lavčević, B. Etlinger, S. Bernstorff, SAXS/DSC study of polymer electrolyte for Zn rechargeable nanostructured galvanic cells. J. Electrochem. Soc. **154**(6), A554-A560 (2007). https://doi.org/10.1149/1.2724440

[S3] L. A. Mary, T. Senthilram, S. Suganya, L. Nagarajan, J. Venugopal, S. Ramakrishna, V. Giri Dev, Centrifugal spun ultrafine fibrous web as a potential drug delivery vehicle. Express Polym. Lett. **7**(3), 238-248 (2013). https://doi.org/10.3144/expresspolymlett.2013.22

[S4] S.-W. Kuo, C.-H. Wu, F.-C. Chang, Thermal properties, interactions, morphologies, and conductivity behavior in blends of poly (vinylpyridine)s and zinc perchlorate. Macromolecules **37**(1), 192-200 (2004). https://doi.org/10.1021/ma035655+

[S5] L.P. Wang, P.F. Wang, T.S. Wang, Y.X. Yin, Y.G. Guo, C.R. Wang, Prussian blue nanocubes as cathode materials for aqueous Na-Zn hybrid batteries. J. Power Sources **355**, 18-22 (2017). https://doi.org/10.1016/j.jpowsour.2017.04.049
